# Agave Wilt Susceptibility by Reduction of Free Hexoses in Root Tissue of *Agave tequilana* Weber var. azul Commercial Plants in the Fructan Accumulation Process

**DOI:** 10.3390/ijms25137357

**Published:** 2024-07-04

**Authors:** Rodrigo Guillermo Mantilla-Blandon, Norma Alejandra Mancilla-Margalli, Joaquín Adolfo Molina-Montes, Jaime Xavier Uvalle-Bueno, Martín Eduardo Avila-Miranda

**Affiliations:** 1 Postgraduate Studies and Research Division, Tecnológico Nacional de México/Instituto Tecnológico de Tlajomulco, Circuito Vicente Fernández-Gómez km 10, Tlajomulco de Zúñiga CP 45640, Jalisco, Mexico; rod.mantilla@gmail.com (R.G.M.-B.); norma.mm@tlajomulco.tecnm.mx (N.A.M.-M.); 2 Postgraduate Studies and Research Division, Tecnológico Nacional de México/Instituto Tecnológico de Tuxtla-Gutiérrez, Carr. Panamericana km 1080, Tuxtla Gutiérrez CP 29050, Chiapas, Mexico; joaquin.mm@tuxtla.tecnm.mx; 3 Research Department, Casa Cuervo México S.A. de C.V., Circunvalación Sur 51-A, Colonia Las Fuentes, Zapopan CP 45070, Jalisco, Mexico; juvalle@cuervo.com.mx

**Keywords:** root rot, sink-source, low sugar disease, fructans polymerization

## Abstract

*Agave tequilana* stems store fructan polymers, the main carbon source for tequila production. This crop takes six or more years for industrial maturity. In conducive conditions, agave wilt disease increases the incidence of dead plants after the fourth year. Plant susceptibility induced for limited photosynthates for defense is recognized in many crops and is known as “sink-induced loss of resistance”. To establish whether *A. tequilana* is more prone to agave wilt as it ages, because the reduction of water-soluble carbohydrates in roots, as a consequence of greater assembly of highly polymerized fructans, were quantified roots sucrose, fructose, and glucose, as well as fructans in stems of agave plants of different ages. The damage induced by inoculation with *Fusarium solani* or *F. oxysporum* in the roots or xylem bundles, respectively, was recorded. As the agave plant accumulated fructans in the stem as the main sink, the amount of these hexoses diminished in the roots of older plants, and root rot severity increased when plants were inoculated with *F. solani*, as evidence of more susceptibility. This knowledge could help to structure disease management that reduces the dispersion of agave wilt, dead plants, and economic losses at the end of agave’s long crop cycle.

## 1. Introduction

Blue agave (*Agave tequilana* Weber var. azul) is the only raw plant material used to produce the internationally recognized spirit beverage tequila. In 2022, 2.611 million metric tons of “heads” (stems) of this plant were used to produce 651.4 million liters of the beverage, according to reports of the Tequila Regulatory Council [1]. Fructans are the main photosynthetic products stored in agave heads [2], and their concentration and degree of polymerization (DP) increase with age [3]. Stored fructans reserve carbohydrates that supply energy for vegetative development [4] and act as osmoprotectants during drought. In agave plants, they must be accumulated before maturity to be mobilized to assemble the floral scape, flowers, and seeds for the sexual reproduction mechanism at the end of its life cycle [5,6]. Maturity and harvest of agave crops take 5 to 8 years, just before flowering, to keep most of the fructan content in the “head”, which is fermented by yeasts and bacteria to obtain alcohol and finally tequila [7,8].

Agave wilt is the most important disease in this crop, characterized by the complete degradation of crown or root tissue or the destruction and/or plugging of vascular bundles, which are symptoms caused by the pathogenic process of *Fusarium solani* (Mart) and *F. oxysporum* (Schlecht) that can finally result in premature death and yield reduction [9,10,11]. Commercial agave fields with a history of agave wilt show a steep rise in incidence and severity when plants increase their head growth rate when they reach 4 to 5 years old. This age coincides with the time when agave plants show an important accumulation of high DP fructans [3,4].

Plant diseases observed at the end of the cycle in other crops such as banana, tomato, cotton, and maize are thought to be the result of “a loss of resistance by a sink” or “low sugar disease” [12,13]. This study aimed to determine whether agave plants increased their susceptibility to agave wilt with age and if this is related to the accumulation of fructans in the stem and the reduction of soluble carbohydrates necessary to mount defense reactions in root tissues.

## 2. Results

### 2.1. Root Rot Severity Caused by F. solani

**Figure 1:**
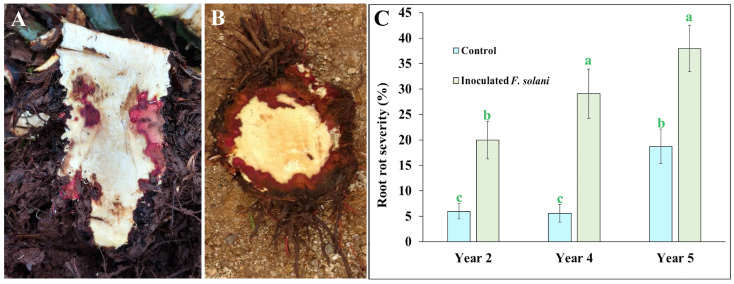
Internal necrotic tissue on the subterranean stem base and crown tissue of 5 years old of *Agave tequilana* (Weber) var. azul plants, inoculated with pregerminated conidia of *Fusarium solani* (Mart) “G” strain. (**A**) Transverse view and (**B**) longitudinal view. (**C**) Root rot severity (%) quantified as length of root necrosis in commercial agave plants of 2, 4, and 5 years old inoculated compared with non-inoculated control plants at field conditions in Acatic, Jalisco, Mexico. Bars with the same letter are statistically equal (LSD test α = 0.05) (error bars = MSE).

Reddish tissue is clear evidence of the necrotrophic pathogenic process induced by strain “G” of *F. solani* [10], and this symptom was observed as massive reddish necrosis at the base of the stem that remained below the soil level or in the crown area in plants inoculated with this fungus (Figure 1A,B).

In the external dark-colored appearance of agave roots, segments with grayish, reddish, or dark brown internally necrotic roots contrasted with the white healthy tissue. The damage quantified as root rot severity in non-inoculated control plants showed that 2- and 4-year-old plants nearly maintained the same amount of root rot severity of around 6%, but in 5-year-old plants, a severity of 18.75% was recorded, which was statistically higher than younger plants. However, agave plants inoculated with pregerminated conidia of *F. solani* showed a root rot severity increase on 2- and 4-year-old plants, displaying a mean of 20% and 29%, respectively, which were statistically higher than non-inoculated control plants of the same age. Older 5-year-old plants reported a significant upsurge, reaching a severity average of 37.9% root rot (Figure 1C).

### 2.2. Histopathology of Damage Caused by F. oxysporum

**Figure 2:**
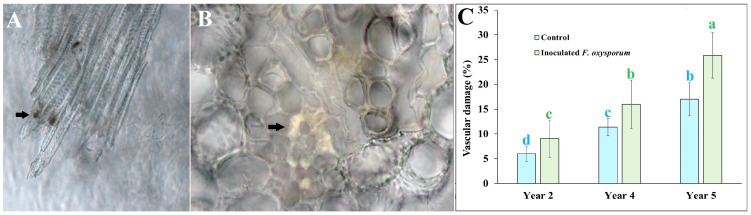
Damage on vascular vessels of *Agave tequilana* (Weber) var. azul plants from a commercial field inoculated with pregerminated conidia of strain SR8 of *Fusarium oxysporum* (Schlecht); (**A**) lateral plane with dark material depositions clogging xylem vessels as indicates with the arrow and (**B**) transversal plane with partial and full clogging as arrow indicates. (**C**) Incidence percentage of clogged and/or deteriorated xylem vessels of plant stems from 2, 4, and 5 years old after two months from inoculation compared to non-inoculated control group. Bars with the same letter are statistically equal (LSD test α = 0.05) (error bars = MSE).

None of the inoculated plants of any age showed foliar wilt symptoms two months after inoculation with pathogenic *F. oxysporum* due to the long incubation period required for the aerial manifestation of symptoms caused by this pathogen. However, microscopic analysis showed stem sections with necrosis or clogged vascular vessels with yellow to reddish-brown materials (Figure 2A,B). The damage shown in inoculated plants is typical of xylem attacked by *F. oxysporum*, as reported in watermelon [14] and blue agave [9].

Xylem damage was also recorded in control non-inoculated plants, with an average ranging from 6% of incidence in 2-year-old plants to 17% in 5-year-old control plants. However, when younger plants were inoculated, the mean xylem vessel obstruction and/or necrosis increased significantly to 9%. In inoculated 5-year-old plants, the increment reached an incidence of 25.85%, significantly more than in non-inoculated plants of the same age (Figure 2C), as seen in the stem incidence data.

### 2.3. Carbohydrates

#### 2.3.1. Water-Soluble Carbohydrate Changes Induced by Necrotrophic *F. solani*

**Figure 3:**
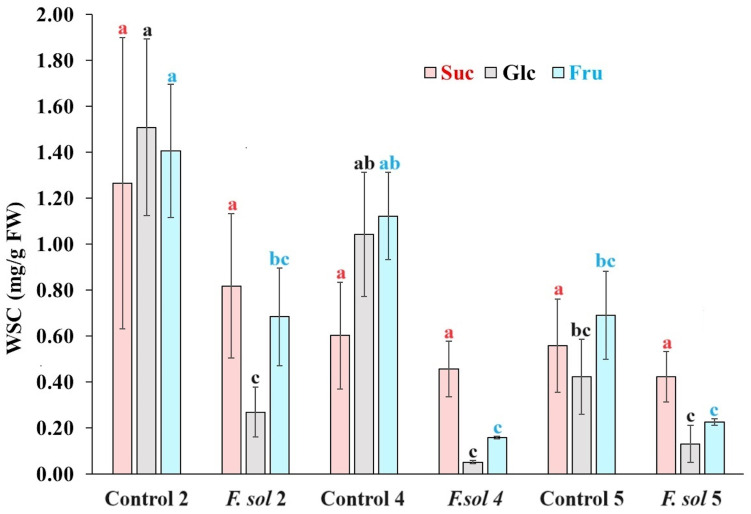
Water-soluble carbohydrate content in root tissue of *Agave tequilana* (Weber) var. azul plants inoculated (*F. sol*) or non-inoculated (control) with pregerminated *Fusarium solani* (Mart) pathogenic “G” strain to agave in field conditions in Acatic, Jalisco, Mexico. Letters above columns show statistical grouping when same carbohydrate is compared. Bars with the same letter are statistically equal (LSD test α = 0.05) (error bars = MSE).

The water-soluble carbohydrate (WSC) content in agave plants interacting with necrotrophic *F. solani* is shown in Figure 3. The sucrose (Suc) content for non-inoculated control plants decreased as they age from 1.27 to 0.56 mg/g FW in 2- and 5-year-old plants, respectively, but without statistical significance. A slight decrease in the mean Suc content was observed after inoculation with *F. solani*; however, these values were not statistically significant.

When the glucose (Glc) content was compared between non-inoculated agave plants, a significant reduction of 1.51 to 0.42 mg/g FW was observed among 2- and 5-year-old plants, respectively. However, when the Glc root content was evaluated after inoculation with *F. solani*, it was significantly reduced in 82% and 95% of inoculated 2- and 4-year-old plants, respectively, when compared with their control plants. The Glc in inoculated 5-year-old plants was similar to the already significantly reduced Glc content in 5-year-old control plants. The Glc of plants inoculated with *F. solani* of the three ages was similar to the Glc concentration of the non-inoculated 5-year-old control plants.

The variation in fructose (Fru) in the non-inoculated control plants was very similar to that observed in their Glc content, considering that the 5-year-old control plants were statistically inferior to the younger 2-year-old plants with 0.69 and 1.41 mg/g FW, respectively. Inoculated 2- and 4-year-old plants had a strong and significant reduction in their Fru concentration with respect to their control plants; however, *F. solani*-inoculated plants of all ages were similar in Fru amount to the statistically reduced 5-year-old control plants.

#### 2.3.2. WSC Changes Induced by Hemibiotrophic *F. oxysporum*

**Figure 4:**
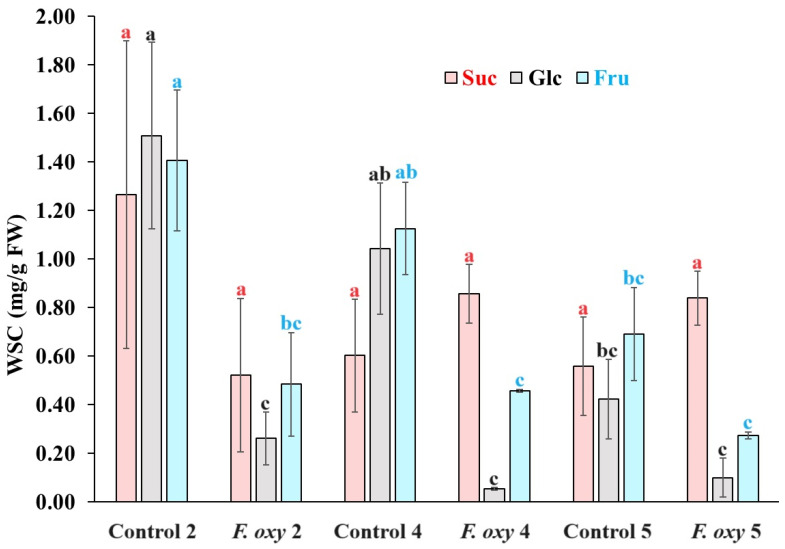
Water-soluble carbohydrate content in root tissue of *Agave tequilana* (Weber) var. azul plants of different ages inoculated (*F. oxy*) or non-inoculated (control) with pregerminated *Fusarium oxysporum* (Schlecht) pathogenic SR8 strain in field conditions in Acatic, Jalisco, Mexico. Letters above columns show statistical grouping when same carbohydrate is compared. Bars with the same letter are statistically equal (LSD test α = 0.05) (error bars = MSE).

Data of root Suc, Glc, and Fru behavior in non-inoculated control plants were the same as those described in the confrontation with *F. solani*, with a significant reduction in the concentration of the two hexoses in 5-year-old plants (Figure 4). When the Suc content was evaluated in *F. oxysporum* inoculated agave plants, an increase in the mean Suc from 0.5213 in 2-year-old plants to 0.8570 and 0.8683 mg/g in 4- and 5-year-old agave plants, respectively, but no statistical significance was determined between inoculated and non-inoculated plants.

When Glc and Fru were determined in *F. oxysporum*-inoculated plants, an important reduction was provoked in the content of these hexoses in 2- and 4-year-old plants compared to their controls; however, these levels in plants of 2-, 4-, and 5-years old were not significantly different from the content of the non-inoculated 5-year-old control plants.

#### 2.3.3. Fructans

**Figure 5:**
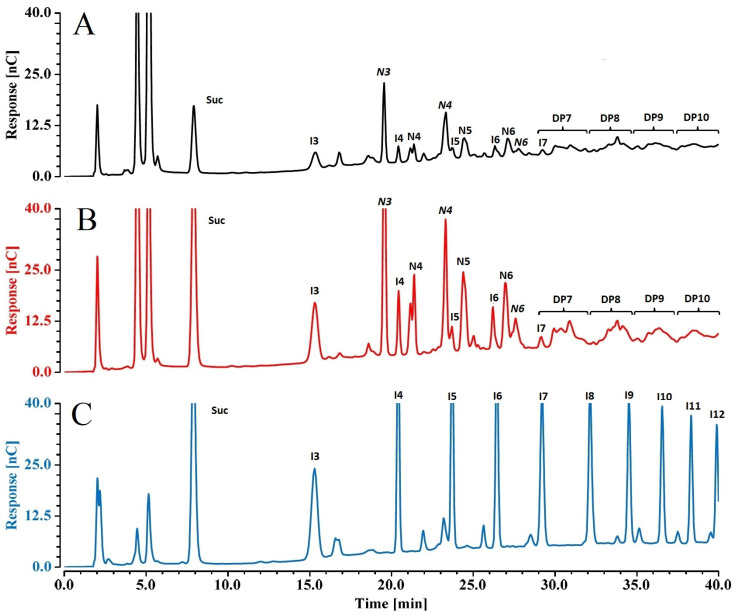
Chromatographic fructan profile of (**A**) 2-year-old *Agave tequilana* (Weber) plant, and (**B**) 5-year-old agave plant, compared to (**C**) fructan (inulin-type) from chicory as standard. Tags above each peak indicate the type of fructan and DP as follows: I3 to I11 inulin (Glc-Frun) from DP3 to DP11; N3 (neokestose, Fru-Glc-Fru), N4 (1,6-Glc-kestotetraose, Fru-Fru-Glc-Fru) and N4 (FGFF, 1&66G-kestotetraose, Fru-Glc-Fru-Fru), DP7 to DP10 include fructans of isomeric structure unresolved under these chromatographic conditions.

Agave is one of 15% of plants that store fructans as reserve carbohydrates; the complex mixture of diverse β-fructosyl–fructose of these polymers contrasts with the homogeneous inulin series in traditional fructan-storage crops, such as chicory and artichoke [2,15]. Figure 5 compares the chromatographic profile of the inulin standard series from chicory with those of water-soluble agave fructans extracted from the stems of younger and older plants. Chicory inulin shows polydisperse fructosyl-β(2-1)–fructose polymers with DP ranging from 3 (Glc-Fru-Fru) to around 11 [Glc-(Fru)_10_]. In addition to the inulin series, agave plants contain other fructan structures, including neofructan and branched (graminan) series; this implies that every Fru added to a previous fructosyl chain generates a higher number of fructan isomers, making their resolution and identification complex. Because commercial standards for agave fructans are not available, the nature of their structures was assigned according to the elution order reported previously [4] and the retention time of each peak for different DP inulin from chicory was used as a reference for the determination of the DP range of different isomers of agave fructans (Figure 4). The amperometric detector is less sensitive as the DP of polysaccharides increases [16]; therefore, fructan content on agave stems was determined semi-quantitatively by comparing the area of every isomer fructan included in each DP range.

Figure 5 shows that neokestose (N3, Fru-Glc-Fru) is in general the most prevalent fructan in agave; this is important in the carbohydrate assembly line, as it is used as a residue for other bigger branched fructans that are polymerized later in the agave life cycle [17]. When the neokestose unit is used for fructosyl moiety addition, two DP4 neofructans are identified: N4 (Fru_2_-Glc-Fru, 1,6-Glc-kestotetraose) and N4 (Fru-Glc-Fru_2_, 1&66G-kestotetraose) [17,18]. The area of fructan peaks at different DPs registered in 2- and 4-year-old plants were statistically similar for inulin DP3 and DP4 (I3 and I4, respectively), and fructans DP6, DP8, and DP9, while 2-year-olds had greater areas than 4-year-olds in the case of N3, DP5, and DP7. Five-year-olds had the highest content in all cases (Table 1).

## 3. Materials and Methods

### 3.1. Chemical Reactives

Potato dextrose agar (PDA) for microbiological procedures was from Difco; eluents for chromatographic analysis were made with 50% NaOH solution (Sigma-Aldrich, Naucalpan de Juárez, Mexico, Ion chromatographic grade, CAS-1310-73-2) and sodium acetate anhydrous from J. T. Baker (CAS 127-09-3). Distilled water was freshly deionized and filtered by nanofilter water system (Barnstead D8991) for 18 MΩ resistivity. Other chemicals used in this work were ACS grade from Merck Sigma Aldrich unless otherwise indicated.

### 3.2. Fusarium Strains

Pathogenic strain “SR8” of *F. oxysporum* and “G” of *F. solani*, previously reported from the *Fusarium* strain collection of the Plant Pathology Laboratory of the Tlajomulco Technological Institute (ITTJ) [9,10], were activated, and pre-germinated conidial suspensions were adjusted to 4 × 10^4^ conidia/mL and used as inoculum.

### 3.3. Agave Plants

**Figure 6:**
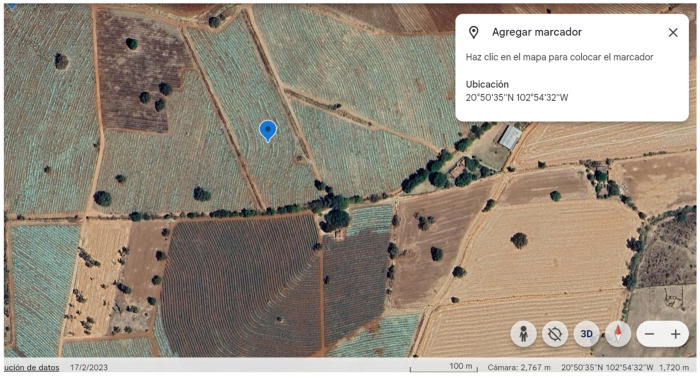
Geographical area where agave plants were cultivated. The marker indicates the zone around which the experiments were carried out. Imagen obtained from Google Earth™ 18 June 2024.

In July 2014, one month after the start of the rainy season, thirty-six agave plants were individually located in three commercial fields established in Santo Domingo, Acatic, Jalisco, Mexico (Figure 6). Groups of twelve of these plants were selected at two, four, and five years from the date of establishment in the field. These plants were selected because of their level “0” on the wilt severity scale of agave [9], meaning healthy plants with no foliar symptoms, with extended leaves, the characteristic bluish-green color of the azul variety in *A. tequilana*, and without dry or curly leaves at the bottom of the plant.

In August 2014, in the middle of the rainy season, four plants of each age were inoculated with 250 mL of the pregerminated conidia of *F. solani* or *F. oxysporum* directly into the soil surrounding the root system of each plant. Four more plants of each age were selected but not inoculated with pathogenic fungi and considered as controls. Plants continued their cycles with typical agronomic management for an additional two months. In October, the plants were dug out for root and stem sampling. The samples were stored at −70 °C until processing.

### 3.4. Disease-Level Evaluation

#### 3.4.1. Severity of Root Rot Caused by *F. solani*

Root fragments (10 cm long) were transversely cut with a scalpel, quantifying the length percentage of rotten tissue, observed as brownish, reddish, or greyish internal tissue, whereas white tissue was considered healthy. Root rot in plants inoculated with strain “G” was quantified as a severity percentage. The percent data were angular transformed before statistical analysis.

#### 3.4.2. Vascular Wilt Injury Caused by *F. oxysporum*

Tissue from the bottom of the stem of each plant was cut off into small cubic fractions with about 0.25 cm sides. From this tissue, freehand cuts were made with a Gillette™ double-edge razor blade and an Iroscope^®^ NZ14B stereoscope at 40×; three thin cuts were obtained from each cubic square. Cuts from tissue of the stem of each plant were stained with lactoglycerol cotton blue and analyzed at 400× with a Carl Zeiss Axiolab^®^ microscope. Digital photographs were taken from different visual fields in each cut with an Axiocam ICc 1 camera and processed with ZEN 2012 blue edition Carl Zeiss microscopy software. Xylem damage caused by the pathogenic process of *F. oxysporum* was quantified as the percentage of incidence of clogged and/or deteriorated vascular vessels in the stem in each visual field and transformed with the square root method for statistical analysis.

### 3.5. Water-Soluble Carbohydrates (WSC)

#### 3.5.1. Extraction

Stem and root tissues were cryofractured in liquid nitrogen and ultrapure water was added to powdered tissue in 1:2 proportion (*w*/*v*) and heated at 95 °C for 10 min for glycosyl hydrolase inactivation. The suspensions were shaken for 10 min and centrifuged at 14,000 rpm for 20 min, and the supernatant was recovered. The extract was then diluted with ultrapure water (18 MΩ resistivity) and filtered with 0.45 μm nylon. Root extracts were screened for Glc and Fru, and fructans were also screened for stem extracts.

#### 3.5.2. Chromatographic Analysis

Carbohydrates were analyzed in a High-Performance Anion Exchange Chromatography system, coupled to a Pulse Amperometric Detector (HPAEC-PAD, Dionex Thermo Scientific™, Sunnyvale, CA, USA), using a Carbo-Pac PA 100 analytical column (2 × 250 mm, 8.5 µm, Thermo Scientific, Sunnyvale, CA, USA) at 30 °C. Glu, Fru, and Suc were eluted with an isocratic mobile phase of 60 mM NaOH at a flow rate of 0.250 mL/min, they were quantified using calibration curves with standard for each molecule (r^2^, 0.9992, 0.9959, and 0.9993 for Glc, Fru, and Suc, respectively).

For fructan analysis, a gradient of water (eluent A), 150 mM NaOH (eluent B), and 500 mM sodium acetate in 150 mM NaOH (eluent C) was used as follows: from 0 to 5 min, 25% of A and 75% of B; from 5 to 40 min, 65% of A and 35% of C. Potentials applied for the amperometric pulse were according to the supplier’s advice.

The distribution of DP of agave inulooligosacharides ranging from 3 to 10 was determined by comparing them with an inulin commercial chicory standard. Both chromatograms of fructans from chicory and agave were graphically compared based on retention times to define the DP in agave samples. However, due to the ramified and more diverse structure of fructans in *A. tequilana*, the total area, in nC/min, in the additional peaks produced between the DP peaks from the chicory lineal inulins was also considered to estimate the fructan content of agave samples in each DP, as reported before [4]. This specific evaluation between DP peaks was also necessary because of the reduction in the HPAEC-PAD detection capacity [16], which can result in the display of smaller peaks in longer retention times for bigger molecules.

### 3.6. Statistical Analysis

All data were analyzed using the GLM procedure and LSD mean test with the software SAS (University Edition, SAS^®^ 3.8 software, Cary, NC, USA), as well as a Pearson correlation analysis when statistical data were suitable.

## 4. Discussion

*F*. *solani*, as a necrotrophic plant pathogen, is capable of using a wide number of virulence strategies to kill plant cells in its post-infection tissue colonization process [10,19]. Excretion of cell wall-degrading enzymes and mycotoxins are among the mechanisms that induce crown and root rot; the level of necrotic plant tissue provoked is directly related to the disease severity, and aerial symptoms include wilting, stunting, and chlorosis [20]. On the other hand, in the plant, the receptors of recognition of molecular patterns associated with pathogens or damage (MAMPs and DAMPs) trigger a series of redundant local or systemic defense mechanisms that include expression of pathogenesis-related proteins (PR), synthesis of phenolics for phytoalexin production, and the cell wall lignification process to increase resistance to *F. solani* enzymatic degradation [21,22,23]. The root system and the stem base are heterotroph sink tissues that depend on the supply of Suc from source tissues in the leaves. The mechanisms of defense used by root cells to stop or reduce their colonization require cell wall invertase activity to increase Glc and Fru hexoses, products of Suc hydrolysis, and to fuel secondary metabolism, which will generate defense compounds to stop the pathogenic process [24,25]. An increase in sugar content in the early stages of infection has been reported to boost plant defense mechanisms in numerous fungi–plant pathosystems [26]. Additionally, high levels of Suc in tolerant tomato genotypes are reported as a key during interaction with *F. solani* for better tolerance to Fusarium wilt [27].

According to this, in this study, the basal Suc and hexose content in the roots was more abundant in younger 2-year-old agave plants than in 5-year-old plants, and the statistically lower severity of root rot after inoculation of healthy plants with *F. solani* likely favored a bigger profile of resistance responses. In agreement with Katagiri [28], defense is energy-intensive, and its limitation can negatively influence the expression of defensive mechanisms. This assumption is additionally supported by the Pearson correlation coefficient between the severity of root rot and the Glc and Fru content in the root tissue of each inoculated agave plant of −0.88 and −0.94, respectively.

Vascular wilt induced by *F. oxysporum* is the product of a more specific pathogenic process realized mainly in the xylem, which starts when penetration hyphae infect roots and colonize xylem vessels, where they proliferate and obstruct ascendent water transport. As the pathogenic process progresses, leaves wilt, eventually causing plant death [29,30,31]. *Fusarium oxysporum* obtains its nutritional requirements mainly from scarce nutrients in xylem sap, and xylem cell walls using enzymatic digestion, invading neighboring cells, or inducing leakage in surrounding tissues [32,33]. After early recognition, plant trays stop or reduce pathogen dispersion in the xylem vessels, producing tyloses, which are outgrowths of contiguous parenchyma cells, to block the spread of pathogens. To better block the vessels, the host plant induces the synthesis of gels rich in pectin that are deposited to seal infected xylem vessels [29,34]. Additionally, parenchyma cells adjacent to xylem vessels produce PR proteins, such as β-1,3-glucanases, chitinases, and peroxidases, which are delivered to the xylem to reduce the dissemination of the pathogen. However, a quick, intense, and durable defense response is necessary to suppress pathogen dispersal, and hexoses are necessary for the tissue actively involved in the resistance response [24,31]. The 2-year-old agave plants had the best nutritional conditions in the roots at the time of infection, which is coincident with Morkunas et al. [35], who reported that during *F. oxysporum* infection, a considerable decrement in WSC post-infection was regularly observed if their content was initially high since this carbon source can be redirected for the defense response, but the reduction is negligible if it is initially low with limited possibilities for defense. Similar behavior of agave plants is supported by the analysis of Pearson correlation coefficients of −0.69 and −0.73 determined between the incidence of xylem injury and Glc and Fru content, respectively.

Plant organs under attack can become a carbohydrate sink to mount an efficient defense strategy [24,36] or for the signaling of different pathways [26], in addition to the costs in terms of resource allocation or more integral parts of the immune system, such as R genes [37]. However, the hexose depletion in root tissue observed over the years in the non-inoculated agave plants shown in this work could indicate that when agave plants reach maturity, the stem is converted as the main storage organ of fructans [3,4]. At this point, the oldest plants were not able to reallocate more carbohydrates to the roots, although they were in urgent need of support to sustain the synthesis of defense mechanisms against soilborne plant pathogens, such as *F. solani* and *F. oxysporum* as is shown in this work.

The increased quantification levels of fructans of DP 10 in 5-year-old plants evidence their accumulation in stems, which is normal at this age in agave plants and a favorable scenario from a productive point of view. This data concurs with Arrizon et al. and Mellado-Mojica and López [3,4] reporting the richest concentration of lower molecular weight fructans in younger 2-year-old agave plants and after that age, a linear relationship between the increment in the fructan content and the plant age occurred in 5-year-old plants.

The high demand for stem fructans as the main sink source is easily supported by a higher foliar area and an efficient photosynthetic capacity [38], this is coincident with the reduction of Suc and free hexoses in root tissue of older plants.

During their life cycles, most plants naturally experience metabolism changes regarding carbon transport and usage in both source and sink organs, as well as in the degree of competition among various sinks for the common pool of carbohydrates available [39,40].

These data show that agave is less susceptible to soilborne plant pathogens, such as *F. solani* and *F. oxysporum*, at a younger age, shifting later to an increased susceptibility when fast fructan accumulation begins and coinciding when the root systems of adjacent plants in the same row start touching each other as they age and grow in commercial fields. This increased susceptibility in older plants explains the common observation of increasing clusters of diseased plants that pass from a healthy status to plants with high agave wilt severity in a few months when they are grown near plants established in hot spots of inoculum that express wilt symptoms early in their crop cycle [10].

These findings can help to improve the management of agave wilt, reducing the quantity or the effectivity of agave wilt inoculum, in those field points where the first young wilt diseased plants were located, using chemical or biological strategies that reduce the increase in epidemics by secondary dispersion from plant to plant in the same row at the end of the crop cycle.

## 5. Conclusions

The data shown in this work confirm the different types of damage caused by two pathogens with different lifestyles. There was a clear trend towards a decrease in the content of Fru and Glc in the roots; this reduction was related to the sudden increase in susceptibility to agave wilt as the plant matures. In contrast, an increase in fructan polymerization in the stem, specifically those of higher DP in older plants, determined until 10 DP by the HPAC-PAD system, indicates a stronger sink.

Knowing this increase in susceptibility when agave plants age, it is possible to locate and sanitize foci of infection, starting from the location of young diseased plants and reducing the secondary spread of the disease. This could reduce the economic losses due to dead plants or plants that were harvested early with less weight to avoid infection.

## Figures and Tables

**Table 1:** Estimation and fructan type identification in stem of *Agave tequilana* Weber var. azul from 2-, 4- and 5-year-olds. Fructan content was estimated according to area under curve of chromatographic peaks (nC/min). Fructan content was compared in the same DP between plants of different ages. Means with the same letter are statistically equal (LSD test α = 0.05).

Fructan Type and DP	2-Year-Old	4-Year-Old	5-Year-Old
I3	0.611 ^ab^	0.521 ^b^	0.778 ^a^
N3	3.623 ^a^	2.427 ^b^	4.652 ^a^
I4	1.416 ^ab^	1.146 ^b^	1.763 ^a^
DP5	0.900 ^a^	0.561 ^b^	1.058 ^a^
DP6	0.950 ^b^	0.834 ^b^	1.349 ^a^
DP7	0.972 ^b^	0.708 ^c^	1.296 ^a^
DP8	0.992 ^a^	0.727 ^b^	1.146 ^a^
DP9	0.784 ^b^	0.649 ^b^	1.233 ^a^
DP10	0.340 ^c^	0.802 ^b^	2.285 ^a^

## Data Availability

The data presented in this study are available on request from the corresponding author.

## References

[1] CTR (2023). Consejo Regulador del Tequila. Información Estadística, Producción Total: Tequila y Tequila 100%. https://www.crt.org.mx/Estadisticas-CRTweb.

[2] Mancilla-Margalli N.A., López M.G. (2006). Water-soluble carbohydrates and fructan structure patterns from *Agave* and *Dasylirion* species. J. Agric. Food Chem..

[3] Arrizon J., Morel S., Gschaedler A., Monsan P. (2010). Comparison of the water-soluble carbohydrate composition and fructan structures of *Agave tequilana* plants of different ages. Food Chem..

[4] Mellado-Mojica E., López M.G. (2012). Fructan metabolism in *A. tequilana* Weber blue variety along its developmental cycle in the field. J. Agric. Food Chem..

[5] Arizaga S., Ezcurra E. (1995). Insurance against reproductive failure in a semelparous plant: Bulbil formation in *Agave macroacantha* flowering stalks. Oecologia.

[6] Escobar-Guzmán R.E., Zamudio-Hernández F., Gil-Vega K., Simpson J. (2008). Seed production and gametophyte formation in *Agave tequilana* and *Agave americana*. Botany.

[7] Aldrete-Tapia J.A., Escalante-Minakata P., Miranda-Castilleja D.E., Hernández-Iturriaga M. (2022). Fermentation conditions for yeast selection and effect of yeast–bacterial interaction in developing a starter culture for tequila fermentation. J. Food Sci..

[8] Ramírez-Guzmán K.N., Torres-León C., Martinez-Medina G.A., de la Rosa O., Hernández-Almanza A., Alvarez-Perez O.B., Araujo R., Rodríguez González L., Londoño L., Ventura J., Alexandru G., Holban A.M. (2019). Traditional fermented beverages in Mexico. Fermented Beverages.

[9] Avila-Miranda M.E., López-Zazueta J.G., Arias-Castro C., Rodríguez-Mendiola M.A., Guzmán de Pena D.A., Vera-Núñez J.A., Peña-Cabriales J.J. (2010). Vascular wilt caused by *Fusarium oxysporum* in agave (*Agave tequilana* Weber var. azul). J. Prof. Assoc. Cactus Dev..

[10] Ramírez-Ramírez M.J., Mancilla-Margalli N.A., Meza-Álvarez L., Turincio-Tadeo R., Guzmán de Pena D., Avila-Miranda M.E. (2017). Epidemiology of Fusarium Agave wilt in *Agave tequilana* Weber var. azul. Plant Prot. Sci..

[11] Rubio-Ríos J.R., Guzmán-Plazola R.A., Ayala-Escobar V., Rubio-Cortés R. (2019). Spatial and temporal dynamics of blue agave (*Agave tequilana* Weber var. azul) wilt in Jalisco Mexico. J. Phytopathol..

[12] Ewané C.A., Nott K., Lassois L., Lepoivre P., de Lapeyre de Bellaire L. (2020). Severe modifications in source-sink ratio influence the susceptibility of bananas to crown rot and its phenolics content. Plant Pathol..

[13] Vanderplank J.E. (1984). Disease Resistance in Plants.

[14] Zhang M., Xu J.H., Liu G., Yao X.F., Li P.F., Yang X.P. (2015). Characterization of the watermelon seedling infection process by *Fusarium oxysporum* f. sp. *niveum*. Plant Pathol..

[15] Praznik W., Löppert R., Cruz-Rubio J.M., Zangger K., Huber A. (2013). Structure of fructo-oligosaccharides from leaves and stem of *Agave tequilana* Weber, var. azul. Carbohydr. Res..

[16] Mechelke M., Herlet J., Benz J.P., Schwarz W.H., Zverlov V.V., Liebl W., Kornberger P. (2017). HPAEC-PAD for oligosaccharide analysis—Novel insights into analyte sensitivity and response stability. Anal. Bioanal. Chem..

[17] Mellado-Mojica E., López-Medina T.L., López M.G. (2009). Developmental variation in *Agave tequilana* Weber var. azul stem carbohydrates. Dyn. Biochem. Biotechnol. Mol. Biol..

[18] Ritsema T., Joling J., Smeekens S. (2003). Patterns of fructan synthesized by onion: Fructan:fructan 6G-fructosyltransferase expressed in tobacco BY2 cells—Is fructan:fructan 1-fructosyltransferase needed in onion?. New Phytol..

[19] Chen L., Wu Q., He T., Lan J., Ding L., Liu T., Wu Q., Pan Y., Chen T. (2020). Transcriptomic and metabolomic changes triggered by *Fusarium solani* in common bean (*Phaseolus vulgaris* L.). Genes.

[20] Coleman J.J. (2016). The *Fusarium solani* species complex: Ubiquitous pathogens of agricultural importance. Mol. Plant Pathol..

[21] Ghozlan M.H., EL-Argawy E., Tokgöz S., Lakshman D.K., Mitra A. (2020). Plant defense against necrotrophic pathogens. Am. J. Plant Sci..

[22] Barros J., Serk H., Granlund I., Pesquet E. (2015). The cell biology of lignification in higher plants. Ann. Bot..

[23] Chávez-Sánchez C., Mancilla-Margalli N.A., Montero-Cortés M.I., Gutiérrez-Miceli F.A., Briceño-Félix G.F., Simpson J.K.W., Avila-Miranda M.E. (2022). Asexually propagated *Agave tequilana* var. azul exhibits variation in genetic markers and defence responses to *Fusarium solani*. AoB Plants.

[24] Pastuszak J., Szczerba A., Dziurka M., Hornyák M., Kopeć P., Szklarczyk M., Płażek A. (2021). Physiological and biochemical response to *Fusarium culmorum* infection in three durum wheat genotypes at seedling and full anthesis stage. Int. J. Mol. Sci..

[25] Proels R.K., Hückelhoven R. (2014). Cell-wall invertases, key enzymes in the modulation of plant metabolism during defence responses. Mol. Plant Pathol..

[26] Morkunas I., Ratajczak L. (2014). The role of sugar signaling in plant defense responses against fungal pathogens. Acta Physiol. Plant..

[27] Alsamir M., Mahmood T., Ahmad N., Trethowan R. (2017). Distribution of organic metabolites after Fusarium wilt incidence in tomato (*Solanum lycopersicum* L.). Aust. J. Crop Sci..

[28] Katagiri F. (2024). A global view of defense gene expression regulation—A highly interconnected signaling network. Curr. Opin. Plant Biol..

[29] Beckman C.H., Tjamos E.C., Beckman C.H. (1989). Colonization of the vascular system of plants by fungal wilt pathogens: A basis for modelling the interaction between host and parasite in time and space. Vascular Wilt Diseases of Plants: Basic Studies and Control.

[30] Rodríguez-Gálvez E., Mendgen K. (1995). The infection process of *Fusarium oxysporum* in cotton root tips. Protoplasma.

[31] Yadeta K.A., Thomma B.P.H.J. (2013). The xylem as battleground for plant hosts and vascular wilt pathogens. Front. Plant Sci..

[32] Divon H.H., Ziv C., Davydov O., Yarden O., Fluhr R. (2006). The global nitrogen regulator, FNR1, regulates fungal nutrition-genes and fitness during *Fusarium oxysporum* pathogenesis. Mol. Plant Pathol..

[33] Rana A., Sahgal M., Johri B.N., Satyanarayana T., Deshmukh S., Johri B. (2017). *Fusarium oxysporum*: Genomics, diversity and plant–host interaction. Developments in Fungal Biology and Applied Mycology.

[34] Agrios G.N. (2005). Plant Pathology.

[35] Morkunas I., Marczak Ł., Stachowiak J., Stobiecki M. (2005). Sucrose-induced lupine defense against *Fusarium oxysporum*. Plant Phys. Biochem..

[36] Kanwar P., Jha G. (2018). Alterations in plant sugar metabolism: Signatory of pathogen attack. Planta.

[37] Karasov T.L., Chae E., Herman J.J., Bergelson J. (2017). Mechanisms to mitigate the trade-off between growth and defense. Plant Cell.

[38] Pimienta-Barrios E., Zañudo-Hernández J., García-Galindo J. (2006). Seasonal photosynthesis in young plants of *Agave tequilana*. Agrociencia.

[39] Valantin M., Gary C., Vaissière B.E., Tchamitchian M., Bruneli B. (1998). Changing sink demand affects the area but not the specific activity of assimilate sources in cantaloupe *Cucumis melo* L.. Ann. Bot..

[40] Osorio S., Ruan Y.L., Fernie A.R. (2014). An update on source-to-sink carbon partitioning in tomato. Front. Plant Sci..

